# Uncovering the sub-lethal impacts of plastic ingestion by shearwaters using fatty acid analysis

**DOI:** 10.1093/conphys/coz017

**Published:** 2019-05-16

**Authors:** Peter S Puskic, Jennifer L Lavers, Louise R Adams, Martin Grünenwald, Ian Hutton, Alexander L Bond

**Affiliations:** 1 Institute for Marine and Antarctic Studies, University of Tasmania, School Road, Newnham, Tasmania, Australia; 2 Institute for Marine and Antarctic Studies, University of Tasmania, 20 Castray Esplanade, Battery Point, Tasmania, Australia; 3 Lord Howe Island Museum, P.O. Box 157, Lord Howe Island, New South Wales, Australia; 4 Bird Group, Department of Life Sciences, The Natural History Museum, Akeman Street, Tring, Hertfordshire, United Kingdom

**Keywords:** Marine debris, nutritional composition, plastic pollution, procellariiform, seabirds

## Abstract

Marine plastic pollution is increasing exponentially, impacting an expanding number of taxa each year across all trophic levels. Of all bird groups, seabirds display the highest plastic ingestion rates and are regarded as sentinels of pollution within their foraging regions. The consumption of plastic contributes to sub-lethal impacts (i.e. morbidity, starvation) in a handful of species. Additional data on these sub-lethal effects are needed urgently to better understand the scope and severity of the plastics issue. Here we explore the application of fatty acid (FA) analysis as a novel tool to investigate sub-lethal impacts of plastic ingestion on seabird body condition and health. Using gas chromatography-mass spectrometry, we identified 37 individual FAs within the adipose, breast muscle and liver of flesh-footed (*Ardenna carneipes*) and short-tailed (*Ardenna tenuirostris*) shearwaters. We found high amounts of FA 16:0, 18:0, 20:5n3 (eicosapentaenoic acid), 22:6n3 (docosahexaenoic acid) and 18:1n9 in both species; however, the overall FA composition of the two species differed significantly. In flesh-footed shearwaters, high amounts of saturated and mono-unsaturated FAs (needed for fast and slow release energy, respectively) in the adipose and muscle tissues were related to greater bird body mass. While total FAs were not related to the amount of plastic ingested in either species, these data are a valuable contribution to the limited literature on FAs in seabirds. We encourage studies to explore other analytical tools to detect these sub-lethal impacts of plastic.

## Teaser

Our study is the first to describe the fatty acid composition of the liver, breast muscle, and adipose tissue from flesh-footed (Ardenna carneipes) and short-tailed shearwaters (A. tenuirostris) and uses this technique to explore possible sub-lethal effects of plastic ingestion on these species.

## Introduction

In <70 years, plastic waste has become persistent in almost all terrestrial and aquatic habitats to such an extent that plastic debris is present in the stratigraphy of most sedimentary deposits and is one of the primary indicators of the human-induced, geological epoch known as the Anthropocene (Waters *et al.*, 2016, Zalasiewicz *et al.*, 2016). Once in marine environments, plastic fragments into small particles known as micro- (1–5 mm) and nano-plastics (<1μm; Barnes *et al.*, 2009, Provencher *et al.*, 2017). Plastic can then be readily consumed by a range of marine life from krill (Dawson *et al.*, 2018) to large marine mammals (Fossi *et al.*, 2018, Nelms *et al.*, 2018). Seabirds, particularly Procellariiformes (i.e. albatross, petrels and shearwaters), have been recorded with some of the highest plastic ingestion rates, which have been attributed to factors such as foraging strategy, plastic colour and odour (Lavers and Bond, 2016, Savoca *et al.*, 2016, Tavares *et al.*, 2017, Verlis *et al.*, 2013). Impacts from the ingestion of plastic may include damage to tissues, morbidity and starvation (Auman *et al.*, 1997, Lavers *et al.*, 2014).

Lipid-derived fatty acids (FAs) serve a functional purpose within an animal’s body where they may be stored or metabolized in a variety of tissues (Parrish *et al.*, 2015). Different FAs serve different metabolic functions in animals and most FAs are combined into a range of lipid classes. These include triglycerides (TAG), which are key to the storage of energy, phospholipids, which comprise the structural components of cell membranes, or wax esters, which are stored in various tissue structures (Ramos and Gonzalez-Solis, 2012). The majority of adipose tissue consists of TAG, which is a crucial energy source for young birds during periods of parental neglect and in preparation for fledging (Ricklefs and Schew, 1994). In migratory birds, adipose tissue containing abundant FAs, such as 16:0 palmitic acid, 18:1n9 (an omega-9 FA) and 18:2n6 (an omega-6 FA), is stored in excess and metabolized rapidly in the extreme energy bursts experienced during migration (McWilliams *et al.*, 2004).

Migratory marine megafauna, which travel large distances to exploit variable food sources, rely heavily on lipid reserves to survive these journeys (Ramos and Gonzalez-Solis, 2012). In some species, adipose deposition has been scored and compared to morphometric measurements to determine body condition (Auman *et al.*, 1997, Cousin *et al.*, 2015, Schultner *et al.*, 2013). Application of these methods to seabirds has received criticism as some measures of condition are thought to be subjective (van Franeker, 2004). Chick growth can be highly variable due to the wide range of prey sources exploited and infrequent feeding of seabird chicks (Angel *et al.*, 2015, Connan *et al.*, 2005, Taylor and Konarzewski, 1989, Williams *et al.*, 2009). As a result, validation of current measures of body condition using direct, precise analytical techniques (e.g. dietary lipids) would inform our understanding of bird health (Schamber *et al.*, 2009). The application of FA analysis of tissues has been used to describe spatial and temporal shifts in diet, revealing trophic interactions (Haynes *et al.*, 2015, Karnovsky *et al.*, 2012). For example, in fledgling seabirds, adipose tissue reflects the dietary FAs consumed by an individual over a period of ~1–2 months (Williams *et al.*, 2009), providing a rapid method to assess short-term diet. The description of FA compositions as a response to toxicological factors, and issues such as the sub-lethal impact of plastic ingestion, has not yet been explored.

Some seabird groups ingest high quantities of plastic (e.g. 56% of Procellariformes; Kühn *et al.*, 2015), which can be a major cause of morbidity and altered physiology (Auman *et al.*, 1997, Lavers *et al.*, 2014); however, very few studies have described this relationship successfully (Carey, 2011, Cousin *et al.*, 2015, Ryan, 1988). The application of FAs may have the potential to explore these important questions in wild, free-living seabirds as current seabird linear morphometric measurements, used as indicators of condition, have not been validated against FA or protein data (Schamber *et al.*, 2009), and these measurements often vary among fledglings within a colony as adults return to feed chicks at different intervals. Here we investigate the relationship between FA composition, linear morphometric measurements and plastic ingestion in two species of pelagic seabird: flesh-footed shearwaters (*Ardenna carneipes*) and short-tailed shearwaters (*Ardenna tenuirostris*) to better understand the potential sub-lethal impacts of ingested plastic.

## Materials and methods

**Figure 1:**
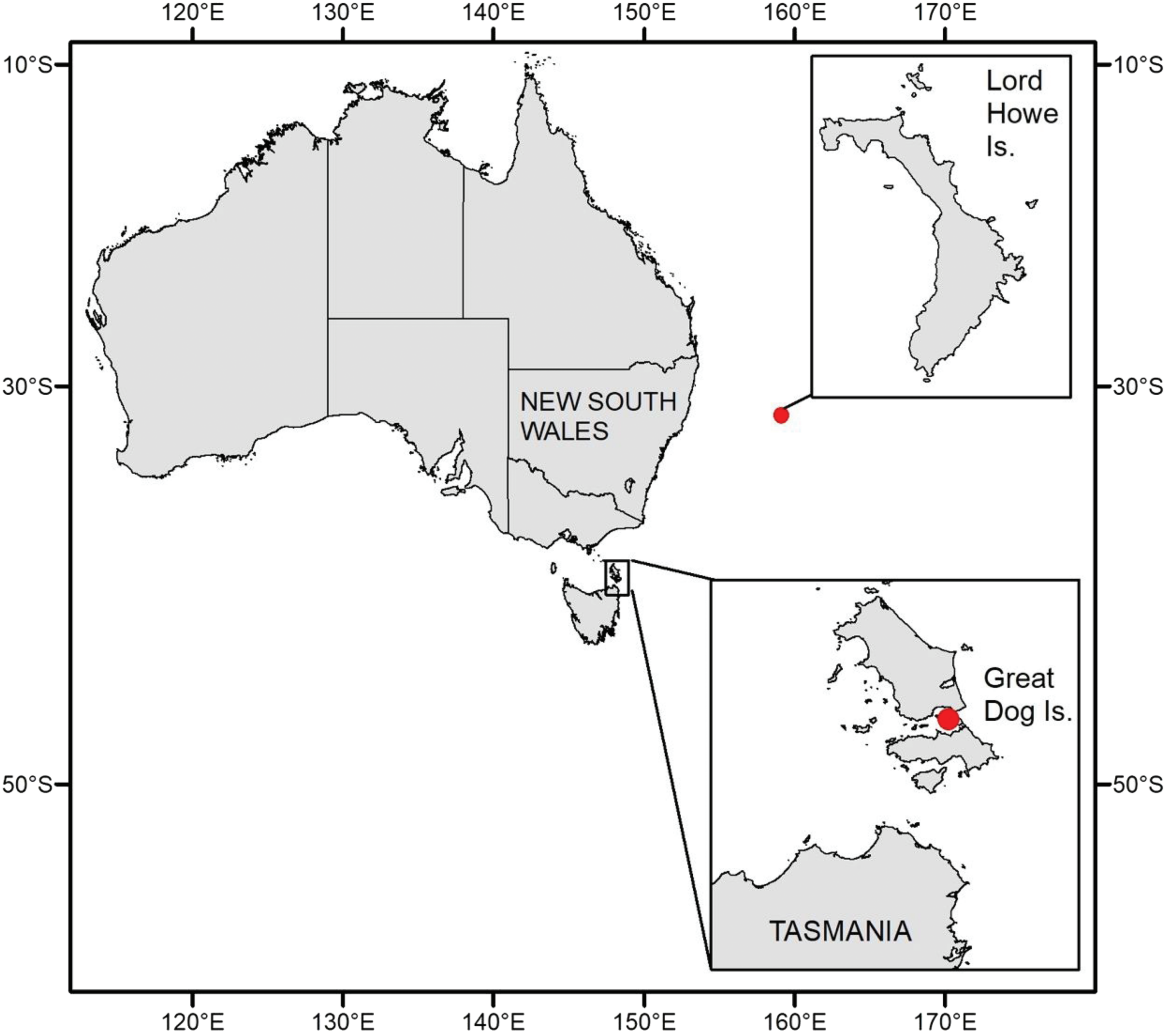
Map of study sites (red dots) shows the location where flesh-footed shearwater fledglings (*A. carneipes*) were sampled from Lord Howe Island, New South Wales, Australia (31.554°S, 159.084°E; top panel) and short-tailed shearwater fledglings (*A. tenuirostris*) were collected from Great Dog Island, Tasmania, (40.247°S, 148.249°E; bottom panel).

We sampled 18 fledgling shearwaters (~80–90 days old) of each species for this study. Freshly dead (road-kill, beach-washed) flesh-footed shearwater fledglings were collected on Lord Howe Island, New South Wales, Australia (31.554°S, 159.084°E) from 26 April to 12 May 2017 (Fig. 1). Short-tailed shearwater fledglings were harvested by local hunters under recreational (individually held) collection permits from Great Dog Island in the Furneaux Group, Tasmania, Australia (40.247°S, 148.249°E) in late April 2017 (Fig. 1). Harvested birds were selected randomly and included a range of body sizes.

Bird body mass (±10 g) was determined using a spring balance, wing chord (unflattened and straightened; ±1 mm) using a stopped ruler, culmen and head + bill length using Vernier callipers (±0.1 mm). Visible ingested plastic items (>1 mm) from the proventriculus and gizzard were dried and weighed to the nearest 0.0001 g using an electronic balance. Partial breast muscle (pectoralis major), one lobe of the liver and 2–5 g subcutaneous adipose tissue from the breast were removed from each bird. Tissues were wrapped in aluminium foil, placed in individually labelled bags and stored at −20°C. Tissues were weighed and then freeze-dried for 72 h for dry matter and percent moisture determination and homogenized using a mortar and pestle. The lipids were extracted using a modified Bligh and Dyer (1959) protocol. Briefly, 0.1 g of the tissue was added to a chloroform/methanol/distilled water (1/2/0.8, v/v/v) solution in a glass flask, shaken multiple times and left overnight for extraction. Phase separation was induced by the addition of chloroform/water (1/1, v/v) and shaking on the following day. The chloroform phase that contained the lipids was concentrated under reduced pressure at 42°C and the total lipid content was determined gravimetrically. Then, 1.5 ml of chloroform was added to the lipid extract and 750 μl (Bligh and Dyer, 1959) was transferred to a screw cap test tube with 100 μl of a surrogate standard working solution, consisting of C:19fatty acid methyl ester (FAME) dissolved in CHCl_3_ (500 mg/l). The mixture was concentrated under a stream of nitrogen gas. Next, 3 ml of saponification reagent (5% (w/v) KOH in 80:20 (v/v) MeOH:H_2_O) was added and the whole solution heated at 80°C for 3 h. Once cooled to room temperature, the mixture was diluted by adding 1 ml of distilled water. To create a phase separation, 1.5 ml of hexane:chloroform (4:1) was added. The top aqueous phase containing the non-saponifiable neutral lipids was removed. The lower layer was mixed by hand with 1 ml 2 M hydrochloric acid and 1.5 ml of hexane chloroform ((Hex:CHCl_3_) 4:1 (v/v)) and the resulting top aqueous layer placed in another vial. Then, 1 ml of methylation reagent was added to this new solution and left to rest for 1 h at 80°C before adding 1 ml H_2_O, and then 1.5 ml of Hex:CHCl_3_ solution was added and the sample concentrated under a nitrogen gas stream. Finally, 1000 μl of internal standard working solution (50 μg/ml C19 FAME in CHCl_3_) was added and the extracted FAME sample was stored at −20°C. Standard working solution was added to each sample at the final step of FAME extraction to calculate the potential sample loss. Blank samples were used to calibrate for outside contaminants during the gas chromatography–mass spectrometry (GC-MS) analysis.

FAME samples were analysed using a Varian CP-3800 gas chromatograph equipped with a CP-8400 autosampler, coupled to a Brüker 300-MS triple quadrupole mass spectrometer. Stationary phase was an Agilent DB-5MS column, 30 m × 0.25 mm, with 0.25 μm phase thickness. Helium was used as the carrier gas. Electron ionization mass spectra of FAME was recorded in full scan mode.

### Statistical analysis

All statistical analysis was completed using R 3.4.3 (R Core Team, 2018) in RStudio (v.1.1.453, Boston, Massachusetts, USA). The relationship between mass of ingested plastic items and bird morphometrics was explored using a linear regression and a Cook’s Distance of > 3 identified statistical outliers (Rousseeuw and Leroy, 2005). Results where *P* < 0.05 were regarded as statistically significant.

Following a similar statistical approach to Williams *et al.* (2009), all FAs that were expressed in the tissues of the individuals at < 0.01% were normalized and the values log-transformed: ${x}_{trans}=\log\ \Big({x}_i/{c}_r\Big)$ where ${x}_i$ denotes the percentage composition of a FA, ${x}_{trans}$ is the transformed FA and ${c}_r$ is a random reference FA found in all samples (in our case, 18:0). Because a log transformation cannot be performed on 0 values, all FA values were altered by adding 0.01; logged values were then analysed using a principal component analysis (PCA). To examine the relationships between FA composition among tissues and species, and in relation to ingested plastic, we used a multivariate analysis of variance (MANOVA) with principle component (PC) scores as the response. A Wilk’s (λ) test statistic was used and relationships were considered significant when *P* < 0.05.

## Results

The mean (±SD) number of plastic items ingested was 4.47 ± 4.71 pieces (range, 0–15 pieces) items weighing 0.0760 ± 0.0784 g (range 0.0000–0.2267 g) for short-tailed shearwaters and 18.44 ± 27.19 pieces (range, 0–116 pieces) items weighing 2.9277 ± 6.4851 g (range, 0.0000–27.4625 g) for flesh-footed shearwaters (Supplementary Table 2 and 3). There was no significant relationship between the mass or number of ingested plastic and body mass (flesh-footed: F_1,15_ = 0.38, *P* = 0.54; short-tailed: F_1,16_ = 0.10, *P* = 0.75), wing chord (flesh-footed: F_1,15_ = 2.24, *P* = 0.16; short-tailed: F_1,16_ = 1.36, *P* = 0.26), head + bill (flesh-footed: F_1,15_ = 0.33, *P* = 0.57; short-tailed: F_1,16_ = 2.85, *P* = 0.11) or culmen length (flesh-footed: F_1,16_ = 0.31, *P* = 0.59; short-tailed: F_1,16_ = 0.37, *P* = 0.55) in either species.

A total of 37 FA were found in each of the three tissues of both species tested (Supplementary Table 1). Mono-unsaturated FA (MUFA) were the predominant FA class, accounting for 35.9% of all FA on average and ranging from 28.9–49.9% in the tissues of short-tailed and from 20.7–37.6% in the tissues of flesh-footed, respectively. The second most abundant FA class were the saturated FAs (SFAs, 31.3–38.1%, 21.8% and 31.2%). The content of the remaining poly-unsaturated FA (PUFA) ranged from 15.9–33.0% and 36.8–57.5% in the two species, respectively.

We identified six abundant FAs that individually accounted for > 5% of the total FA composition in both short-tailed and flesh-footed shearwaters, respectively; 16:0 (19.1–23.8%, 19.4–25.5%), 18:0 (4.5–16.8%, 10.5–12.1%), 16:1b (0.0–7.9%, 0.0–5.3%), 22:6n3 (6.2–13.8%, 4.2–7.3%), 20:5n3 (4.3–8.68%, 1.7–2.9%) and 18:1n9 (19.8–29.5%, 26.2–33.0%).

**Figure 2:**
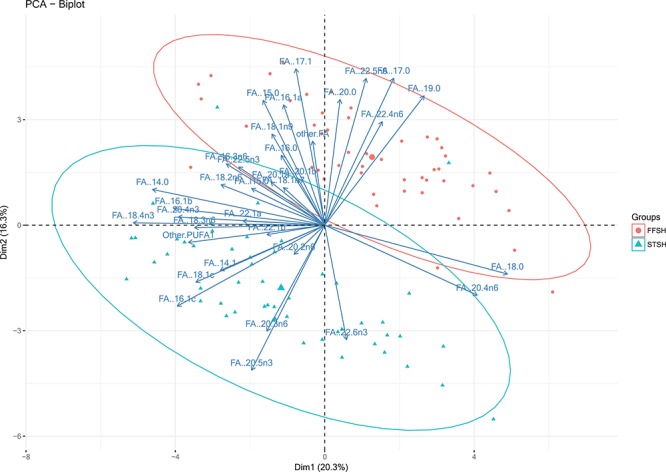
Biplot of PC1 (Dim1) and 2 (Dim2) of the PCAs of soft tissue samples (adipose, breast muscle and liver) from short-tailed shearwaters (STSH; blue triangles) and flesh-footed shearwater (FFSH; red circles). FA compositions were normalized and the values log transformed. Any FA composition value over 0.01% was included as a loading on the PCA. A coloured version of this figure is available online.

A PCA was run on all FAs detected in the samples (Fig. 2). The factor loadings for PCs 1–4 accounted for 62% of the variance in the data. The two species grouped distinctly with little overlap between the FA compositions (Fig. 2). PC 1 and 3 were strongly driven by low percentages of FAs 16:1, 18:1 and 18:2n6 while PC 2 and 4 were strongly positively correlated with low proportions of FAs 15:0, 17:0, 17:1, 20:5n3 and 22:6n3 FAs (Table 1).

MANOVA analysis indicated that body mass was positively related to MUFAs and SFAs (Table 1) in the adipose (F_4,5_ = 5.04, *P* = 0.053, λ = 0.801) and breast muscle tissue (F_4,6_ = 25.57, *P* < 0.001, λ = 0.944) of flesh-footed shearwaters. However, there was no statistically significant association with FAs and body mass in the liver tissue (F_4,9_ = 2.25, *P* = 0.14, λ = 0.49). Short-tailed shearwaters showed associations between FAs and body mass in the liver (F_4,8_ = 4.14, *P* = 0.042, λ = 0.674) and muscle tissue (F_4,9_ = 4.61, *P* = 0.027, λ = 0.672). This was not present in adipose (F_4,8_ = 0.70, *P* = 0.61, λ = 0.73).

**Table 1:** Factor loadings of each of the first four PCs of the PCA of 34 detected short-tailed (*A. tenuirostris*) and flesh-footed (*A. carneipes*) shearwater FAs including MUFAs and PUFA.

FA	PC1	PC2	PC3	PC4
				
14:1	−0.18	−0.09	0.07	−0.09
14:0	−0.29	0.07	−0.23	0.05
i15:0	−0.12	0.07	−0.01	−0.06
15:0	−0.11	0.25	−0.26	−0.12
16:0	−0.07	0.14	−0.35	−0.05
17:0	0.12	0.30	0.08	−0.23
18:0	0.31	−0.10	0.11	−0.14
19:0	0.17	0.26	0.16	−0.05
20:0	0.03	0.25	0.23	0.09
16:1a	−0.07	0.24	−0.03	−0.12
16:1b	−0.26	0.03	−0.16	0.02
16:1c	−0.25	−0.16	0.11	−0.04
17:1	−0.05	0.32	−0.07	0.04
18:1c	−0.22	−0.12	0.08	0.16
18:1n9	−0.09	0.18	0.12	0.27
18:1n7	−0.07	0.08	0.22	−0.26
20:1a	−0.09	0.09	0.07	−0.09
20:1b	−0.05	0.09	0.31	0.13
22:1a	−0.14	0.01	0.33	0.22
22:1b	−0.10	−0.02	0.38	0.18
18:2n6	−0.18	0.08	0.11	−0.32
20:2n6	−0.05	−0.06	0.06	−0.07
16.3n6	−0.17	0.12	−0.05	−0.04
18:3n6	−0.22	0.00	0.06	−0.01
20:3n6	−0.10	−0.21	0.21	−0.24
18:4n3	−0.33	0.00	−0.07	−0.01
20:4n6	0.26	−0.14	−0.01	−0.30
20:4n3	−0.25	0.02	0.13	−0.23
22:4n6	0.10	0.21	0.18	−0.13
20:5n3	−0.13	−0.29	−0.08	−0.25
22:5n6	0.07	0.30	0.03	−0.18
22:5n3	−0.15	0.12	0.15	−0.33
22:6n3	0.04	−0.23	−0.02	−0.24
Other MUFA	−0.02	0.17	−0.19	−0.04
Other PUFA	−0.23	−0.03	0.04	−0.04

Neither species showed any relationship between the mass of plastic ingested and FA compositions (PC 1–4) in adipose (flesh-footed: F_4,5_ = 1.12, *P* = 0.41, λ = 0.49; short-tailed: F_4,8_ = 0.76, *P* = 0.57, λ = 0.28) or liver tissues (flesh-footed: F_4,9_ = 0.24, *P* = 0.91, λ = 0.09; short-tailed: F_4,8_ = 1.16, *P* = 0.39, λ = 0.37). Breast muscle in short-tailed shearwaters showed a relationship between FAs and mass of plastic ingested (F_4,9_ = 5.15, 0.019, λ = 0.696). Flesh-footed shearwaters did not (F_4,6_ = 1.03, *P* = 0.46, λ = 0.41).

## Discussion

### Differences in FA compositions

Palmitic acid (16:0) is the most abundant FA found in animals, and is commonly the product of *de novo* synthesis of 14-carbon FAs within the liver of seabirds (Käkelä *et al.*, 2009). This particular FA was the most abundant in the tissues of both shearwater species examined (Supplementary Table 1), as well as in the breast muscle of the closely related sooty shearwater (*Ardenna grisea*) (Al-Amer *et al.*, 2016) and stomach oil of sub-Antarctic breeding albatrosses (Connan *et al.*, 2014). Palmitic acid may be stored in the adipose tissue or used rapidly as an energy substrate (Williams and Buck, 2010). The abundance of this FA is believed to be important for the release of energy during migration, as observed in songbirds (McWilliams *et al.*, 2004) and may explain why it is found in such high levels in fledgling shearwaters.

Essential fatty acids (EFAs) must be obtained through diet and cannot be synthesized *de novo*. The EFAs 20:5n3 (eicosapentaenoic acid, EPA) and 22:6n3 (docosahexaenoic acid, DHA) were found in high concentrations in the liver and muscle tissues of both species (Supplementary Table 1). EPA was found in relative abundance in short-tailed shearwaters (6.5% ± 2.7%) compared to flesh-footed shearwaters (2.2% ± 1.2%) and other members of the Procellariidae (Al-Amer *et al.*, 2016, Wang *et al.*, 2007, Woodward *et al.*, 1995). Overall, EFAs are crucial for avian health, including the development of polar lipid structures (i.e. muscle) in young birds (as has been shown in chickens; Maldjian *et al.*, 1995).

This study demonstrates the scope of intra-genus physiological differences among the two study species of shearwaters. Differences in FA composition among tissue types can likely be attributed to the function of the tissues (e.g. the liver in bioprocessing and *de novo* synthesis of FAs), the turnover rate of FAs within certain tissues and the typical lipid classes that are associated with each tissue type (Ramos and Gonzalez-Solis, 2012, Williams and Buck, 2010). Results from the PCA (Fig. 2) indicate flesh-footed and short-tailed shearwaters form two distinct groups based on their FA composition. The two species feed in different environments on prey from low trophic, sub-Antarctic environments (short-tailed shearwaters) or high trophic, sub-tropical environments (flesh-footed shearwaters); therefore, the FA composition of prey species is likely driving this difference, since the majority of FAs is derived from prey (Connan *et al.*, 2005). Flesh-footed shearwaters feed primarily on mesopelagic fishes and squid (Gould *et al.*, 1997), prey that are typically high in 16:0, 18:1n9 and 22:6n3 FAs (Lewis, 1967). Conversely, short-tailed shearwaters forage on smaller, lower trophic level prey, with Antarctic krill (*Euphausia superba*) and small cephalopods being the most dominant species (Carey, 2011, Connan *et al.*, 2005). Antarctic krill are abundant in 14:0, 16:0, 16:1 and 20:5n3 FAs (Fricke *et al.*, 1984), and this may be reflected in the FA outputs of these birds (Supplementary Table 1). We found higher amounts of 18:1n9 in flesh-footed shearwater tissues (29.4% ± 6.8%) and more 20:5n3 in short-tailed shearwaters (6.5% ± 2.7%), supporting the diet-driven differences above. While Procellariiformes exhibit some variation in their FA composition (Al-Amer *et al.*, 2016, Wang *et al.*, 2007, Woodward *et al.*, 1995), all exhibit high levels of palmitic acid, 18:0 (stearic acid) and DHA. The variance in FAs among species is likely the result of differences in diet, as well as nutritional physiology and life history. Both DHA and stearic acid are most common in marine prey species (Speake *et al.*, 1999). From the limited information available, the FA composition of flesh-footed and short-tailed shearwaters, though individually different, is consistent with that of other Procellariiformes.

### Body condition and plastic ingestion

FA PC scores and linear morphometrics showed no relationship with the mass of plastic ingested in either species, except in the muscle of short-tailed shearwaters (*P <* 0.019*)*. Analysis of flesh-footed shearwater data from 2011 suggested a strong link between plastic ingestion and decreased body size at fledging, and potentially a poorer likelihood of survivability due to high plastic ingestion (Lavers *et al.*, 2014). The same analysis conducted on birds from 2017 failed to show these relationships, serving as a reminder of the annual variability in plastic ingestion (much lower in 2017 compared to 2011), in populations’ responses to ingestion, and the difficulty of identifying sub-lethal impacts of plastic in seabirds. Finally, our inability to detect a relationship between ingested plastic, linear morphometrics and FA composition across both species may have been hampered by the small sample size (*n* = 18 in 2017 compared to *n* = 37 in 2011) and variability in the number and mass of plastic ingested by birds in 2017. The mean mass of ingested plastic was unrelated to body size of short-tailed shearwaters collected in 2017, a pattern that was also reported by Carey (2011) and Cousin *et al.* (2015) who failed to find statistically significant relationship between plastic and fat condition scores. Plastic ingestion was, however, related to short-tailed shearwater FA composition in the breast muscle only, indicating plastic at this concentration either had few measurable impacts on this species, or that impacts affected different physiological pathways.

It is possible this study may have overlooked a number of dietary-derived FAs in tissues with fast metabolic turn over, as the fledgling birds collected at the time of this study were undergoing a period of parental neglect and may not have fed in 1–2 weeks (Schultz and Klomp, 2000, Williams *et al.*, 2007). This period of short starvation may also influence the variability in these results. Allmann *et al.* (1965) observed spikes in 16:1 and 18:1 FAs in liver samples after re-feeding starved rats (*Rattus norvegicus*) and suggested a relationship with linoleic acid (18:2n6) deficiencies. Our birds showed high concentrations of 16:1 FA, which may align with our knowledge of their life history (i.e. periods of parental neglect). Few data are available on FA profiles in relation to starvation in seabirds (Phillips and Hamer, 1999). FA metabolism in seabirds differs significantly from other species, including mammals, thereby limiting the application of this literature (Williams and Buck, 2010). More seabird-focused studies are required to develop a comprehensive understanding of these relationships.

Bird body mass and the abundance of subcutaneous adipose tissue have been used widely as an indicator of body condition in free-living birds (Williams *et al.*, 2009). Connors and Smith (1982) was one of the earliest studies to identify a negative correlation between ingested plastic and fat deposition in seabirds. Auman *et al.* (1997) continued to explore fat scoring as an indicator for condition, a method that has been criticized for being subjective (van Franeker, 2004). This presents an opportunity to identify alternate techniques to determine body condition and justify morphometric measurements in this group of seabirds. Schamber *et al.* (2009) argued that a quantitative index of condition is required to limit similar criticisms and provide confidence when using condition indices to assess health. Using PCA to explore FA composition, we determined PC1 and PC3 in the adipose and muscle of flesh-footed shearwaters and liver and muscle of short-tailed shearwaters were correlated with bird body mass (Table 1). This suggests FAs can accurately reflect certain morphometric measurements when sampled from the correct tissues, information that can be used to justify these measurements in future studies.

## Conclusions

Effective conservation and management of seabirds relies on a comprehensive and robust understanding of the pressures faced by populations and individuals. Current knowledge of the impacts of marine plastic pollution is limited to superficial health assessments and documentation of debris ingestion by species. FA analysis was one tool to explore how plastic may disrupt nutritional pathways, though our findings suggest that at least with our sample of birds from a single year, there was no effect. Other similar tools may play an important role in uncovering the sub-lethal impacts of plastic, and should be explored as current conservation and management strategies are not able to account for morbidity and mortality associated with the less visible impacts of plastic. As plastic production and waste increases, we expect to see these impacts on wildlife increase.

## Supplementary Material

Puskic_etal_-_Supplementary_Data_20190221_coz017
