# Supplementary material for: Uncovering the sub-lethal impacts of plastic ingestion by shearwaters using fatty acid analysis
Source: Conserv Physiol. 2019 May 16;7(1):coz017. doi: 10.1093/conphys/coz017 (PMC6521682; doi:10.1093/conphys/coz017)
Supplement: Puskic_etal_-_Supplementary_Data_20190221_coz017 [file puskic_etal_-_supplementary_data_20190221_coz017.docx]

**Supplementary Material:**

Exploring the sub-lethal effects of plastic ingestion by shearwaters using fatty acid analysis

Peter S. Puskic^1^, Jennifer L. Lavers^2*^, Louise R. Adams, Martin Grünenwald^1^, Ian Hutton^3^, Alexander L. Bond^2,4^

*Corresponding author: Jennifer L. Lavers ([Jennifer.lavers@utas.edu.au](mailto:Jennifer.lavers@utas.edu.au), +613 6324 3868), Institute for Marine and Antarctic Studies, University of Tasmania, 20 Castray Esplanade, Battery Point, Tasmania 7004, Australia

**Supplementary Table 1.** Comparison of mean percentage Fatty Acid (FA) composition and standard deviation (± SD), including the total Saturated Fatty Acids (SFA), Monunsaturated (MUFA) and Polyunsaturated Fatty Acids (PUFA), between the soft tissues of fledgling short-tailed (*Ardenna tenuirostris*) and flesh-footed (*A. carneipes*) shearwaters collected in 2017.

|  | Short-tailed Shearwater  (Great Dog Island, Tasmania) | | | | | | | | | | |  | Flesh-footed Shearwater  (Lord Howe Island, New South Wales) | | | | | | | | | | |  |
| --- | --- | --- | --- | --- | --- | --- | --- | --- | --- | --- | --- | --- | --- | --- | --- | --- | --- | --- | --- | --- | --- | --- | --- | --- |
|  | Adipose | | |  | Liver | | |  | Breast muscle | | |  | Adipose | | |  | Liver | | |  | Breast muscle | | |  |
|  | Mean |  | SD |  | Mean |  | SD |  | Mean |  | SD |  | Mean |  | SD |  | Mean |  | SD |  | Mean |  | SD |  |
| 14:0 | 4.78 | ± | 1.63 |  | 1.30 | ± | 1.07 |  | 2.23 | ± | 0.93 |  | 1.81 | ± | 1.48 |  | 1.40 | ± | 1.47 |  | 3.19 | ± | 2.69 |  |
| 15:0 | 0.31 | ± | 0.07 |  | 0.17 | ± | 0.06 |  | 0.23 | ± | 0.05 |  | 0.46 | ± | 0.23 |  | 0.33 | ± | 0.23 |  | 0.61 | ± | 0.29 |  |
| 16:0 | 23.81 | ± | 1.94 |  | 19.07 | ± | 2.90 |  | 19.97 | ± | 2.91 |  | 24.51 | ± | 3.78 |  | 19.35 | ± | 6.25 |  | 25.52 | ± | 6.89 |  |
| 17:0 | 0.29 | ± | 0.06 |  | 0.34 | ± | 0.07 |  | 0.40 | ± | 0.28 |  | 1.01 | ± | 0.30 |  | 0.84 | ± | 0.30 |  | 0.89 | ± | 0.28 |  |
| 18:0 | 4.54 | ± | 1.32 |  | 16.80 | ± | 4.44 |  | 8.15 | ± | 2.56 |  | 11.94 | ± | 5.22 |  | 12.17 | ± | 6.15 |  | 10.52 | ± | 6.00 |  |
| 19:0 | 0.00 | ± | 0.02 |  | 0.01 | ± | 0.03 |  | 0.03 | ± | 0.06 |  | 0.23 | ± | 0.17 |  | 0.30 | ± | 0.17 |  | 0.15 | ± | 0.13 |  |
| 20:0 | 0.22 | ± | 0.09 |  | 0.11 | ± | 0.11 |  | 0.15 | ± | 0.12 |  | 0.44 | ± | 0.28 |  | 0.66 | ± | 0.48 |  | 0.24 | ± | 0.08 |  |
| 22:0 | 0.00 | ± | 0.01 |  | 0.07 | ± | 0.11 |  | 0.01 | ± | 0.03 |  | 0.05 | ± | 0.08 |  | 0.16 | ± | 0.17 |  | 0.01 | ± | 0.03 |  |
| ∑SFA | 34.20 | ± | 2.29 |  | 38.08 | ± | 3.14 |  | 31.33 | ± | 5.38 |  | 31.18 | ± | 18.45 |  | 25.57 | ± | 18.22 |  | 21.83 | ± | 22.04 |  |
| 14:1 | 0.06 | ± | 0.07 |  | 0.01 | ± | 0.03 |  | 0.25 | ± | 0.13 |  | 0.01 | ± | 0.03 |  | 0.03 | ± | 0.05 |  | 0.06 | ± | 0.10 |  |
| 16:1a | 0.29 | ± | 1.04 |  | 0.00 | ± | 0.00 |  | 0.03 | ± | 0.05 |  | 0.14 | ± | 0.15 |  | 0.24 | ± | 0.29 |  | 0.14 | ± | 0.14 |  |
| 16:1b | 7.86 | ± | 2.50 |  | 2.80 | ± | 1.50 |  | 6.29 | ± | 1.53 |  | 3.86 | ± | 1.66 |  | 3.10 | ± | 2.17 |  | 5.29 | ± | 3.43 |  |
| 16:1c | 0.28 | ± | 0.18 |  | 0.14 | ± | 0.16 |  | 0.27 | ± | 0.19 |  | 0.06 | ± | 0.10 |  | 0.02 | ± | 0.04 |  | 0.03 | ± | 0.06 |  |
| 17:1 | 0.22 | ± | 0.13 |  | 0.02 | ± | 0.05 |  | 0.11 | ± | 0.16 |  | 0.40 | ± | 0.24 |  | 0.23 | ± | 0.19 |  | 0.38 | ± | 0.27 |  |
| 18:1c | 0.58 | ± | 0.21 |  | 0.21 | ± | 0.16 |  | 0.32 | ± | 0.14 |  | 0.07 | ± | 0.09 |  | 0.11 | ± | 0.07 |  | 0.04 | ± | 0.07 |  |
| 18:1n7 | 4.09 | ± | 0.71 |  | 3.41 | ± | 0.66 |  | 5.47 | ± | 0.62 |  | 4.37 | ± | 1.04 |  | 4.03 | ± | 1.62 |  | 4.12 | ± | 0.77 |  |
| 18:1n9 | 29.53 | ± | 2.44 |  | 19.80 | ± | 3.52 |  | 29.49 | ± | 1.77 |  | 30.28 | ± | 5.20 |  | 33.00 | ± | 8.04 |  | 26.22 | ± | 6.83 |  |
| 20:1a | 3.44 | ± | 2.06 |  | 0.91 | ± | 0.65 |  | 2.68 | ± | 1.19 |  | 2.73 | ± | 2.22 |  | 7.67 | ± | 6.44 |  | 2.41 | ± | 1.33 |  |
| 20:1b | 0.94 | ± | 1.55 |  | 0.55 | ± | 0.90 |  | 0.86 | ± | 1.76 |  | 0.38 | ± | 0.26 |  | 0.51 | ± | 0.37 |  | 0.22 | ± | 0.22 |  |
| 22:1a | 1.60 | ± | 0.83 |  | 0.41 | ± | 0.35 |  | 0.72 | ± | 0.21 |  | 0.65 | ± | 0.63 |  | 1.80 | ± | 2.38 |  | 0.23 | ± | 0.22 |  |
| 22:1b | 0.53 | ± | 0.31 |  | 0.21 | ± | 0.19 |  | 0.43 | ± | 0.14 |  | 0.28 | ± | 0.30 |  | 0.63 | ± | 0.68 |  | 0.14 | ± | 0.14 |  |
| 22:1c | 0.08 | ± | 0.09 |  | 0.01 | ± | 0.03 |  | 0.01 | ± | 0.02 |  | 0.07 | ± | 0.16 |  | 0.11 | ± | 0.12 |  | 0.06 | ± | 0.11 |  |
| 24:1a | 0.12 | ± | 0.10 |  | 0.07 | ± | 0.14 |  | 0.05 | ± | 0.07 |  | 0.02 | ± | 0.05 |  | 0.10 | ± | 0.16 |  | 0.05 | ± | 0.17 |  |
| 24:1b | 0.21 | ± | 0.14 |  | 0.32 | ± | 0.42 |  | 0.14 | ± | 0.11 |  | 0.17 | ± | 0.26 |  | 0.57 | ± | 0.56 |  | 0.05 | ± | 0.10 |  |
| ∑MUFA | 49.92 | ± | 2.74 |  | 28.90 | ± | 5.06 |  | 47.15 | ± | 1.92 |  | 31.42 | ± | 21.18 |  | 37.68 | ± | 26.62 |  | 20.77 | ± | 21.54 |  |
| 18:2n6 | 2.24 | ± | 0.37 |  | 1.40 | ± | 0.49 |  | 2.21 | ± | 0.53 |  | 1.57 | ± | 0.46 |  | 1.34 | ± | 0.40 |  | 1.69 | ± | 0.71 |  |
| 20:2n6 | 0.00 | ± | 0.00 |  | 0.00 | ± | 0.00 |  | 0.01 | ± | 0.04 |  | 0.00 | ± | 0.00 |  | 0.00 | ± | 0.00 |  | 0.00 | ± | 0.00 |  |
| ∑PUFA2 | 2.24 | ± | 0.37 |  | 1.40 | ± | 0.49 |  | 2.23 | ± | 0.53 |  | 1.13 | ± | 0.82 |  | 0.97 | ± | 0.70 |  | 0.89 | ± | 1.00 |  |
| 16:3n6 | 0.05 | ± | 0.07 |  | 0.00 | ± | 0.02 |  | 0.00 | ± | 0.00 |  | 0.01 | ± | 0.04 |  | 0.03 | ± | 0.06 |  | 0.01 | ± | 0.04 |  |
| 18:3n6 | 0.10 | ± | 0.05 |  | 0.01 | ± | 0.02 |  | 0.03 | ± | 0.04 |  | 0.03 | ± | 0.05 |  | 0.04 | ± | 0.08 |  | 0.02 | ± | 0.05 |  |
| 20:3n6 | 0.15 | ± | 0.05 |  | 0.42 | ± | 0.16 |  | 0.24 | ± | 0.08 |  | 0.03 | ± | 0.05 |  | 0.05 | ± | 0.11 |  | 0.01 | ± | 0.02 |  |
| ∑PUFA3 | 0.31 | ± | 0.15 |  | 0.44 | ± | 0.17 |  | 0.26 | ± | 0.09 |  | 0.02 | ± | 0.07 |  | 0.02 | ± | 0.06 |  | 0.11 | ± | 0.34 |  |
| 16:4n3 | 0.00 | ± | 0.00 |  | 0.00 | ± | 0.00 |  | 0.00 | ± | 0.00 |  | 0.09 | ± | 0.08 |  | 0.11 | ± | 0.11 |  | 0.12 | ± | 0.13 |  |
| 18:4n3 | 0.81 | ± | 0.30 |  | 0.14 | ± | 0.15 |  | 0.24 | ± | 0.20 |  | 0.12 | ± | 0.17 |  | 0.12 | ± | 0.24 |  | 0.20 | ± | 0.26 |  |
| 20:4n3 | 0.37 | ± | 0.13 |  | 0.24 | ± | 0.08 |  | 0.37 | ± | 0.12 |  | 0.26 | ± | 0.18 |  | 0.25 | ± | 0.23 |  | 0.32 | ± | 0.21 |  |
| 20:4n6 | 0.31 | ± | 0.07 |  | 7.41 | ± | 2.00 |  | 1.62 | ± | 0.54 |  | 3.42 | ± | 2.68 |  | 3.08 | ± | 3.03 |  | 4.83 | ± | 4.27 |  |
| 22:4n6 | 0.03 | ± | 0.12 |  | 0.03 | ± | 0.04 |  | 0.02 | ± | 0.04 |  | 0.16 | ± | 0.15 |  | 0.18 | ± | 0.10 |  | 0.25 | ± | 0.31 |  |
| 20:5n3 | 4.32 | ± | 1.50 |  | 8.68 | ± | 2.80 |  | 6.82 | ± | 1.89 |  | 2.00 | ± | 0.68 |  | 1.75 | ± | 1.31 |  | 2.93 | ± | 1.62 |  |
| 22:5n3 | 0.71 | ± | 0.12 |  | 0.70 | ± | 0.14 |  | 0.95 | ± | 0.33 |  | 1.06 | ± | 0.57 |  | 0.88 | ± | 0.70 |  | 0.95 | ± | 0.48 |  |
| 22:5n6 | 0.28 | ± | 1.14 |  | 0.01 | ± | 0.04 |  | 0.00 | ± | 0.00 |  | 0.21 | ± | 0.15 |  | 0.14 | ± | 0.11 |  | 0.17 | ± | 0.15 |  |
| 22:6n3 | 6.21 | ± | 1.95 |  | 13.80 | ± | 3.45 |  | 8.83 | ± | 2.57 |  | 6.61 | ± | 3.72 |  | 4.27 | ± | 2.51 |  | 7.33 | ± | 4.37 |  |

∑values (i.e. ∑SFA) include unidentified FAs.

**Supplementary Table 2.** Percentage of ingested plastic by colour and type recovered from fledgling short-tailed (*Ardenna tenuirostris*) and flesh-footed (*A. carneipes*) shearwaters collected in 2017.

| Species | **Colour** | | | | | |  | **Type** | | | | | |
| --- | --- | --- | --- | --- | --- | --- | --- | --- | --- | --- | --- | --- | --- |
|  | **White** | **Blue** | **Green** | **Red** | **Yellow** | **Black** |  | **Nurdle/**  **Pellet** | **Foam** | **Thread** | **Sheet** | **Fragment** | **Other** |
| Short-tailed Shearwater | 65.91 | 3.41 | 17.05 | 0.00 | 0.00 | 13.64 |  | 18.18 | 0.00 | 1.14 | 1.14 | 79.55 | 0.00 |
| Flesh-footed Shearwater | 83.23 | 3.35 | 3.35 | 3.66 | 1.52 | 4.88 |  | 5.49 | 0.00 | 0.00 | 0.00 | 94.21 | 0.30* |

*****A white balloon was recovered inside the proventriculus of one bird

**Supplementary Table 3.** Distribution of plastic in the digestive tracts of short-tailed (*Ardenna tenuirostris*) and flesh-footed (*A. carneipes*) shearwater fledglings in 2017. FO = frequency of occurrence (%)

| Species |  | Proventriculus | | | |  |  | Gizzard | | | |
| --- | --- | --- | --- | --- | --- | --- | --- | --- | --- | --- | --- |
|  | Mean mass  (g) | | Mean number of items | Range | FO ( |  | Mean mass  (g) | | Mean number of items | Range | FO ( |
|  |  |  |  |  | %) |  |  |  |  |  | %) |
| Short-tailed Shearwater | 0.0164 ± 0.255 | | 1.00 ± 1.60 | 0 - 5 | 20.45 |  | 0.0597 ± 0.0750 | | 4.12 ± 4.63 | 0 - 13 | 79.55 |
| Flesh-footed Shearwater | 3.0145 ± 6.4342 | | 14.29 ± 26.88 | 0 - 113 | 73.64 |  | 0.0841 ± 0.0905 | | 5.12 ± 5.66 | 0 - 19 | 26.36 |
